# TSPO Ligand-Methotrexate Prodrug Conjugates: Design, Synthesis, and Biological Evaluation

**DOI:** 10.3390/ijms17060967

**Published:** 2016-06-18

**Authors:** Valentino Laquintana, Nunzio Denora, Annalisa Cutrignelli, Mara Perrone, Rosa Maria Iacobazzi, Cosimo Annese, Antonio Lopalco, Angela Assunta Lopedota, Massimo Franco

**Affiliations:** 1 Department of Pharmacy–Pharmaceutical Sciences, University of Bari “Aldo Moro”, Bari 70125, Italy; nunzio.denora@uniba.it (N.D.); annalisa.cutrignelli@uniba.it (A.C.); mara.perrone@uniba.it (M.P.); rosamaria.iacobazzi@uniba.it (R.M.I.); angelaassunta.lopedota@uniba.it (A.A.L.); massimo.franco@uniba.it (M.F.); 2 Istituto tumori IRCCS “Giovanni Paolo II”, Flacco, St. 65, Bari 70124, Italy; 3 Department of Chemistry, University of Bari “Aldo Moro”, Bari 70125, Italy; cosimo.annese@uniba.it; 4 Department of Pharmaceutical Chemistry, University of Kansas, Lawrence, KS 66047, USA; lopalco@ku.edu

**Keywords:** translocator protein, methotrexate, TSPO-ligand, bio-conjugate, glioma

## Abstract

The 18-kDa translocator protein (TSPO) is a potential mitochondrial target for drug delivery to tumors overexpressing TSPO, including brain cancers, and selective TSPO ligands have been successfully used to selectively deliver drugs into the target. Methotrexate (MTX) is an anticancer drug of choice for the treatment of several cancers, but its permeability through the blood brain barrier (BBB) is poor, making it unsuitable for the treatment of brain tumors. Therefore, in this study, MTX was selected to achieve two TSPO ligand-MTX conjugates (TSPO ligand α-MTX and TSPO ligand γ-MTX), potentially useful for the treatment of TSPO-rich cancers, including brain tumors. In this work, we have presented the synthesis, the physicochemical characterizations, as well as the *in vitro* stabilities of the new TSPO ligand-MTX conjugates. The binding affinity for TSPO and the selectivity *versus* central-type benzodiazepine receptor (CBR) was also investigated. The cytotoxicity of prepared conjugates was evaluated on MTX-sensitive human and rat glioma cell lines overexpressing TSPO. The estimated coefficients of lipophilicity and the stability studies of the conjugates confirm that the synthesized molecules are stable enough in buffer solution at pH 7.4, as well in physiological medium, and show an increased lipophilicity compared to the MTX, compatible with a likely ability to cross the blood brain barrier. The latter feature of two TSPO ligand-MTX conjugates was also confirmed by *in vitro* permeability studies conducted on Madin-Darby canine kidney cells transfected with the human MDR1 gene (MDCK-MDR1) monolayers. TSPO ligand-MTX conjugates have shown to possess a high binding affinity for TSPO, with IC_50_ values ranging from 7.2 to 40.3 nM, and exhibited marked toxicity against glioma cells overexpressing TSPO, in comparison with the parent drug MTX.

## 1. Introduction

**Figure 1:**
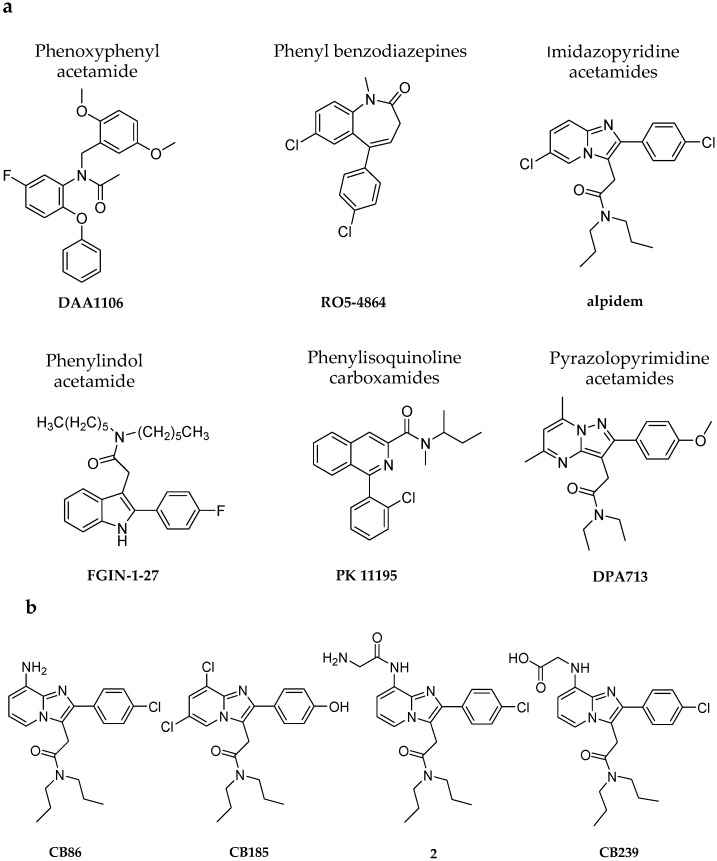
Chemical structures of the typical synthetic TSPO ligands presented in structural classes (**a**); and selective TSPO ligands with hydrophilic substituents (**b**).

The subcellular 18-kDa translocator protein (TSPO) [1], is an attractive biomarker for molecular imaging and a potential therapeutic target for drug delivery to tumors overexpressing TSPO [2,3]. TSPO is mostly located in the outer mitochondria membrane of steroid-synthesizing cells in peripheral organs system. In contrast, its presence in the central nervous system is delimited to ependymal cells including the glia. TSPO is involved in several pathophysiological processes, such as steroidogenesis, immunomodulation, apoptosis, brain injury, neurodegeneration, and cancer [4,5,6,7]. TSPO is upregulated in neuroinflammation and its overexpression has been proved in several types of cancers, including gliomas, whereas expression in the healthy brain is low [8,9]. Thus, TSPO could serve as potential therapeutic tool to target brain tumors using TSPO-ligands conjugates with anti-cancer drugs. Classical synthetic TSPO ligands include phenylisoquinoline carboxamides (e.g., PK 11195), benzodiazepines (e.g., RO5-4864), phenoxyphenyl acetamide (e.g., DAA1106), pyrazolo[1,5-*a*]pyrimidine acetamides (e.g., DPA-713), indoleacetamides (FGIN-1-27), and imidazo[1,2-*a*]pyridines (e.g., alpidem) [5,10] (Figure 1a). Recently, we have synthesized new 2-phenylimidazo[1,2-a]pyridine acetamides, designed from alpidem by introducing hydrophilic substituents on the imidazopyridine nucleus, that bind the mitochondrial protein with high affinity and selectivity (Figure 1b) [10]. The structure-activity analysis has shown that the substitution at the 8-position of the imidazopyridine nucleus with appropriate lipophilic or hydrophilic groups, combined with a chlorine atom added at the *para*-position of the phenyl ring are key factors to increase the affinity and the selectivity toward the TSPO binding sites [10]. Additionally, the incorporation of amino-, hydroxyl-, or carboxylic groups allows for convenient bioconjugation with anticancer drugs.

Methotrexate (MTX; 2,4-diamino-*N*^10^-methylpteroylglutamic acid; **1**) is an anticancer drug of choice for the treatment of several cancers such as acute lymphocytic leukemia, choriocarcinoma, non-Hodgkin’s lymphoma, gastric, breast, head, and neck cancers [11]. MTX and its active metabolites (polyglutamates) are competitive inhibitors of the enzyme dihydrofolate reductase (DHFR) that lead to blockage of tetrahydrofolate synthesis and the depletion of nucleotide precursors. MTX is a class IV drug in the FDA’s Biopharmaceutical Classification System (BCS, Amidon, cFederal Drug and Food Administration, Rockville, MD, USA) with a low permeability (see Table 1) and poor aqueous solubility [12]. It has a short plasma half-life and poor permeability across blood-brain barrier (BBB), when used in normal dosages of the protocols, making it unsuitable to brain tumors [13]. The use of high-dose MTX alone was proposed as the first-line treatment for primary central nervous system (CNS) lymphoma, but the side effects were more obvious. In fact, the myelosuppression, a common side effect and meningitis that often appears in the intrathecal treatment are dose-dependent and are more severe in patients receiving MTX in high dose. Moreover, CNS toxicities, including acute encephalopathy, also occur when the drug is used in high doses [13]. For these reasons, MTX is a suitable candidate for this study and therefore it was selected as a model drug to achieve TSPO ligand-MTX conjugates, potentially useful for the treatment of TSPO-rich cancers, including brain tumors overexpressing the TSPO. In this paper, we describe the synthesis, the physicochemical characterization, as well as the *in vitro* stabilities of the new TSPO ligand-MTX conjugates. The binding affinity for TSPO and selectivity *versus* the central-type benzodiazepine receptors (CBR) of conjugates were also evaluated. Furthermore, the cytotoxicity of prepared compounds was investigated on human SF126 and SF188, and rat RG2 and C6 glioma cell lines, together with their ability to permeate MDCK-MDR1 cell monolayers.

## 2. Results and Discussion

**Scheme 1:**
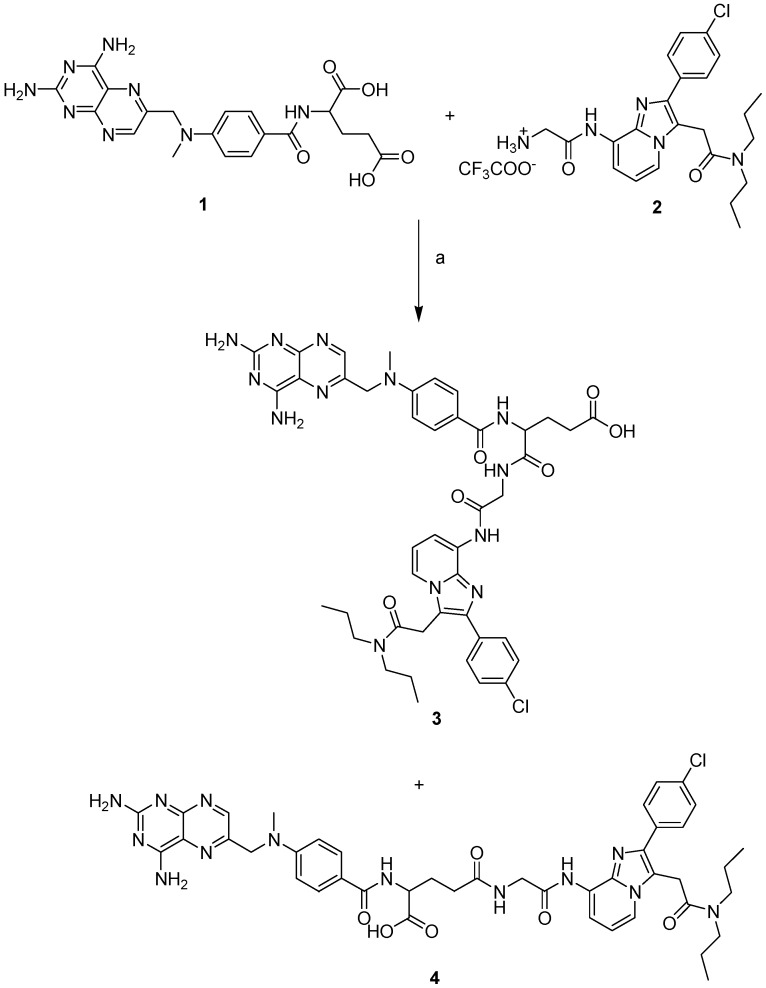
Synthesis of TSPO-ligand α-MTX (methotrexate) conjugate **3** and TSPO-ligand γ-MTX conjugate **4**. Reagents and conditions: (a) 1,1′-carbonyldiimidazole (CDI), anhydrous *N*,*N*-dimethylformamide DMF, room temperature.

A drug delivery system, such as a bio-conjugate that carries an anticancer drug to brain tumors overexpressing the mitochondrial protein, should have favorable biopharmaceutical properties that include membrane permeability, high receptor binding affinity and selectivity, cytotoxicity or ability to convert itself into a cytotoxic moiety. The conjugate strategy is widely implemented to optimize biopharmaceutical and pharmacokinetic characteristics of drugs, including the transport across biological barriers and the reduction of adverse side effects [14]. Usually, the design of a conjugate involves the formation of a covalent chemical bond between the drug and a pharmacologically-inactive portion (e.g., a backbone polymer), whilst activation occurs after *in vivo* administration (conjugate prodrugs) or after reaching the target site upon cellular internalization. The further development of the conjugate is the strategy of the bioconjugate, which contributes in the direct linkage of the drug to a pharmacologically-active portion (e.g., a selective ligand or a peptide), or by the intermediacy of a spacer. In this regard, a number of bioconjugates with selective TSPO ligands have been developed for molecular imaging or for the delivery of hydrophilic anticancer drugs into brain tumors across the BBB. Bioconjugation of nanodevices with TSPO ligands (bio-conjugates with low molecular weight, TSPO decorated nanoparticles, and TSPO-targeted dendrimers) have also been described [15,16,17,18]. Moreover, in our previous studies we pointed out that some selective TSPO-ligands showed apoptotic effects and that the simultaneous transport of a TSPO-ligand with an anticancer drug may result in synergistic effects, precisely the synergism of the antitumor activity of the anticancer drug and of the TSPO ligand [16]. Thus, the aim of this study was to synthesize two new bio-conjugates of the anticancer drug MTX with the potent and highly selective TSPO ligand **2** (Scheme 1).

### 2.1. Chemistry

Conjugation of MTX (non-hydrated form and with unprotected carboxylic groups) with TSPO ligand **2** as trifluoroacetic acid salt was performed in anhydrous *N*,*N*-dimethylformamide (DMF) at room temperature, using carbonyldiimidazole (CDI) as the coupling agent. Under these conditions, TSPO-ligand α-MTX conjugate **3** and TSPO-ligand γ-MTX conjugate **4** (Scheme 1), could be obtained in 70:30 ratio (α/γ).

**Figure 2:**
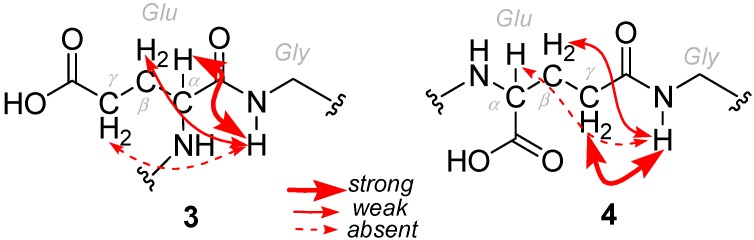
Relevant nuclear overhauser effect spectroscopy (NOESY) correlations for TSPO-ligand α-/γ-MTX conjugates (**3** and **4**, respectively).

The TSPO-ligand MTX conjugates were completely characterized by spectroscopic techniques and mass spectrometry. The ESI-HRMS spectra showed a peak at *m*/*z* = 876.3459 (TSPO-ligand MTX conjugate **3**, [M − H]^−^) or at 876.3430 (TSPO-ligand MTX conjugate **4**, [M − H]^−^), both in agreement with the expected chemical formula, C_43_H_48_ClN_13_O_6_. Additionally, the one-dimensional (1D-) and two-dimensional (2D-) nuclear magnetic resonance (NMR) characterization (^1^H, correlation spectroscopy (COSY), and nuclear overhauser effect spectroscopy (NOESY)) provided spectra in full agreement with the structures assigned to **3** and **4**. The interpretation of combined 2D spectra can prove extremely useful in discriminating the structure of regioisomers [19]. In the case at hand, the NOESY spectra of the two regioisomers **3** and **4** show major differences in the intensity of cross-peaks occurring between the Gly NH of the TSPO moiety and the protons of Glu side-chain. Figure 2 summarizes these relevant NOESY correlations. In one case, the strong NH/α-CH correlation and the weak NH/β-CH_2_ are consistent with conjugation of TSPO ligand to the α-COOH of MTX. In the other case, the absence of the NH/α-CH correlation and the strong NH/γ-CH_2_ correlation are distinctive features of the conjugation to the γ-COOH of MTX.

### 2.2. Lipophilicity

The lipophilicity of a molecule, quantitatively expressed as LogP can be useful to predict its permeability through biological barriers. The lipophilicity of TSPO-ligand **2**, MTX and of the TSPO-ligand MTX conjugates **3** and **4** was estimated by calculating their 1-octanol/water partition coefficients, using CLOGP software (Toronto, ON, Canada), based on the fragmental method of Hansch and Leo [20]. Compounds that possess a value of LogP ≥2.5 are able to cross the BBB. As it can be seen from data in Table 1, both TSPO-ligand MTX conjugates have logP values higher than +2.5 (*i.e.*, +5.21 for **3** and +4.40 for **4**), and appeared to be suitably lipophilic to cross the BBB. Conversely, the parent drug has a negative value of CLogP (−0.24), in line with the experimental evidence (as mentioned above) that MTX is not able to cross the BBB effectively.

### 2.3. Stability Studies

The chemical stability of the TSPO-ligand MTX conjugates **3** and **4** was evaluated in 50 mM phosphate buffer solution at pH 7.4 and 37 °C; the physiological stability was also determined using a dilute rat serum solution (50% *v*/*v*) at 37 °C. The half-lives were calculated by the disappearance of the starting conjugate and are reported in Table 1. The aim of this work is the synthesis of TSPO ligand-MTX conjugates in order to improve the plasma stability and having less side effects than MTX. In addition TSPO ligand-MTX conjugates could enhance the transport across BBB and due a tumor targeting effect for the TSPO moiety, might selectively deliver the antineoplastic agent to brain tumors and prolong its efficacy. The TSPO-ligand MTX conjugates **3** and **4** are stable in buffer solution at pH 7.4 and enough stable in physiological medium with their half-lives exceeding 3 h. The conjugates show a similar stability in serum while TSPO-ligand α-MTX conjugate **3** is the most stable in phosphate buffer with a doubled half-life to the TSPO-ligand γ-MTX conjugate **4**. The liquid chromatography mass spectrometry (LC-MS) (ESI^+^) analysis of the degradation mixture, obtained from enzymatic kinetics samples after 2 h, shows that the degradation of the conjugates occurs through the hydrolysis of the amide bond leading to free MTX (*m*/*z* = 477, [M + Na]^+^) and TSPO ligand **2** (*m*/*z* = 464, [M + Na]^+^). In addition, CB86 (*m*/*z* = 407, [M + Na]^+^) (Figure 1), most likely originating from the degradation of ligand **2**, as previously shown [18], could be detected. Other products arising from the further degradation of the anticancer drug were found.

### 2.4. Radioligand Binding Assays

Binding affinities of the TSPO-ligand MTX conjugates **3** and **4** for TSPO and central-type benzodiazepine receptor (CBR) were assessed on membrane preparations from rat cerebral cortex. The IC_50_ values were determined from the displacement curves using the reference compound [^3^H]-PK 11195, for TSPO, or [^3^H]flunitrazepam for CBR. The data obtained, expressed as IC_50_ values, as well as the ratios between CBR and TSPO affinity, respectively (Selectivity Index, SI), as a measure of the *in vitro* selectivity, are shown in Table 2 and were compared with unlabeled TSPO selective ligand PK 11195, and with CBR selective ligand flunitrazepam. The results of the binding affinities indicate that TSPO-ligand MTX conjugates **3** and **4** show high affinity for TSPO, with IC_50_ values ranging from 7.2 to 40.3 nM. Moreover, the SI of TSPO-ligand MTX conjugate **3** is similar to the reference compound PK 11195. Radioligand binding assays on human brain tissue showed a genetic polymorphism and the variation of binding affinity of selective ligands for the TSPO, due to presence of differing binders in the general population (*i.e.*, high-affinity binders, HABs, low-affinity binders, LABs and mixed-affinity binders, MABs) [21]. Thus, the binding quantification of TSPO ligands is confounded by the expression of different forms of TSPO and to assess the potentially therapeutic use of TSPO-ligands would be appropriate to genetically test the patients.

### 2.5. Cytotoxicity Studies

Cytotoxicity assays of the TSPO-ligand MTX conjugates **3** and **4** were conducted against SF126 and SF188 human glioma cells and, RG2 and C6 rat glioma cells and results are also shown in Table 2. These cellular lines were selected for their high expression level of TSPO and, therefore, have been used extensively for *in vitro* experiments on the brain tumor models and for cytotoxicity studies relating to anticancer drugs, as well as TSPO-ligands and its nanodevices. The tested compounds show high cytotoxicity on all cell lines, especially for C6 rat glioma cells. In particular, the TSPO-ligand MTX α-conjugates **3** appears to be the most cytotoxic displaying a lower IC_50_ value compared to MTX on C6 cells.

### 2.6. Transport Studies

Transport studies of the TSPO-ligand MTX conjugates **3** and **4** were carried out on a MDCKII-MDR1 cell monolayer and results are shown in Table 2. This approach is an established and versatile *in vitro* method to evaluate the drug permeability through the BBB, as well MDCKII-MDR1 was identified as the most promising cell line for qualitative predictions of brain distribution [22]. However He and co-worker have reported the brain microvascular endothelial cells (BMECs) as a more established approach to address the permeability through the BBB [23]. This method was previously described by Audus and has been also used in our recent work [24,25].

The apparent permeability coefficient (*P*_app_) of each compound was calculated on the basis of the amounts permeated through MDCKII-MDR1 monolayer by the equation:

$$P_{\mathrm{app}}=\frac{V_{A}\;dC}{A{\left[D\right]}_{\text{in}}\;dt}$$

where V_A_ (*dC/dt*) is the linear appearance rate of mass in the receiver solution; A is the filter/cell surface area and [D]_in_ is the initial tested compound concentration in the apical (AP) chamber. The *P*_app_ values were evaluated in apical to basolateral direction. Moreover, the flux of fluorescein isothiocyanate-dextran (FD4, Sigma, Milano, Italy) (200 µg/mL) and diazepam (75 µM) as paracellular and transcellular markers, respectively, was measured as an internal control to verify the cell monolayer’s integrity and tight junction integrity during the assay. On an experimental day, the integrity of the cell monolayer was assessed by measuring the transendothelial electrical resistance (TEER) value. The TEER of the BBB was assessed close to 200 Ω/cm^2^, precluding the access to a wide number of common anticancer drugs to brain tissue, including MTX. The results of the transport studies demonstrate that both TSPO-ligand MTX conjugates **3** and **4** were characterized by higher *P*_app_ values than MTX and paracellular marker FD4 (3.54 ± (0.39 × 10^−10^)), with a *P*_app_ values being about. 30 times greater than the parent drug. Moreover, the permeability of MTX-conjugates was found to be lower than that of the transcellular marker diazepam (6.40 ± (0.31 × 10^−5^)). Finally, these results demonstrate that although the MTX-conjugates possessed an adequate calculated lipophilic character, which result in a better permeation capacity, compared to the MTX, the *P*_app_ values were predictive of a modest ability to overcome the BBB by the transcellular pathway. However, due to the development of brain tumors, the structure of tumor vasculature changes and the BBB is gradually replaced by the more permeable blood-brain tumor barrier (BBTB) [26].

## 3. Experimental Section

### 3.1. Materials and Methods

Anhydrous *N*,*N*-dimethylformamide (DMF), 1,1′-carbonyldiimidazole (CDI), *N*-(3-dimethylaminopropyl)-*N′*-ethylcarbodiimide hydrochloride (EDAC), 2,4-diamino-*N*^10^-methylpteroylglutamic acid (methotrexate, MTX), dichloromethane (CH_2_Cl_2_), hexadeuterodimethyl sulfoxide (DMSO-*d*_6_), l-glutamine, penicillin (100 U/mL), and streptomycin (100 µg/mL) were purchased from Sigma (Milan, Italy). Reagent grade chemicals were used without further purification. TSPO ligand, *N*,*N*-dipropyl-[2-(8-(2-aminoacetamido)-2-(4-chlorophenyl)imidazo[1,2-a]pyridin-3-yl)]acetamide (**2**) was synthesized as trifluoroacetate salt, in accord to the procedures described previously [18]. Melting points were determined in open capillary tubes on a Büchi apparatus and are uncorrected. Elemental analyses were performed on a Eurovector elemental analyzer EA 3000. Electrospray mass spectrometry (ESI-MS) were carried out with an Agilent 1100 LC-MSD trap system instrument (Agilent, Santa Clara, CA, USA). IR spectra were obtained using a FT-IR spectrophotometer (Perkin-Elemer, Waltham, MA, USA) using KBr pellets. ^1^H-NMR, COSY, and NOESY NMR spectra were recorded with an Agilent (VNMRS500) 500 MHz spectrometer (Agilent). In the experimental section the values of the coupling constant, *J*, are given in hertz (Hz) and ^1^H chemical shifts are reported as δ values (*i.e.*, in parts per million, ppm), relative to the residual isotopic impurity of DMSO-*d*_6_ solvent (2.50 ppm). The following codes are used for the description of signal multiplicity: singlet (s), doublet (d), triplet (t), doublet of doublets (dd), multiplet (m), br (broad signal), Ar (aromatic H). Silica gel 60 (70–230 mesh, (Merck, Darmstadt, Germania)) was used for column chromatography. Reactions were performed under an argon atmosphere.

### 3.2. High-Performance Liquid Chromatography (HPLC) Analyses

HPLC analyses were performed with an Agilent 1260 Infinity Quaternary LC System equipped with an Agilent variable wavelength UV detector, a Rheodyne injector (Rheodyne, Model 7725i, Agilent) equipped with a 20 µL loop and a OpenLAB CDS ChemStation software (Agilent). A reversed phase Symmetry C18 column (25 cm × 3.9 mm; 5 µm particles) as the stationary phase and a mixture of methanol and deionized water 80:20 (*v*/*v*) as the mobile phase with the flow rate of 1.0 mL/min were utilized and the column effluent was monitored continuously at 254 nm. The compounds were quantified by measuring the areas of the peaks, and using, as references, suitable standard solutions, chromatographed under the same conditions. The data were processed using Microsoft Excel 2010 or GraphPad Software (La Jolla, CA, USA).

### 3.3. Stability Studies in Phosphate Buffer Solution

TSPO ligand-MTX conjugates **3** and **4** chemical stabilities were studied at pH 7.4 in 0.05 M phosphate buffer at 37 °C. The kinetic studies were carried out by adding 100 µL of a stock solution of the conjugate (1.0 mg/mL in DMSO) to 1.9 mL of the buffer solution preheated at 37 °C. Thus, the final concentration of the compounds in the tested solutions was 1 M. The resulting solutions were vortexed and maintained in a shaker water bath at the constant temperature of 37 °C (±0.2). At appropriate time intervals, aliquots of 100 µL of each sample were removed, filtered through cellulose acetate membranes (0.22 µm, Advantec MFS, Pleasanton, CA, USA), and then 20 µL of filtrates were immediately analyzed by HPLC. The studies were done in triplicate and the pseudo-first-order rate constants were obtained from the slopes of the linear plot of the logarithms of the residual concentrations of conjugates against time.

### 3.4. Stability Studies in Rat Serum Solution

The TSPO ligand-MTX conjugates **3** and **4** stabilities in physiological medium were studied at 37 °C in 0.05 M phosphate buffer and 0.14 M NaCl at pH 7.4, containing 50% *v*/*v* of rat serum. The *in vitro* stabilities were assayed by adding 100 µL of the stock solution of conjugates in DMSO (1 mg/mL) to 1.1 mL of preheated serum solution, and the mixture was maintained in water bath at 37 °C (±0.2). Thus, the final concentration of the compounds in the tested solutions was 1 M. At different time points over a period of 0–120 min, aliquots of 100 µL of seach sample were withdrawn and added to 500 µL of cold acetonitrile for deproteinize the serum. Samples were centrifuged at 3500 rpm for 10 min, and the supernatant was removed, filtered through cellulose acetate membranes (0.22 µm, Advantec MFS) and then 20 µL of filtrates were immediately analyzed by HPLC. The studies were done in triplicate and the pseudo-first-order rate constants were obtained from the slopes of the linear plot of the logarithms of the residual concentrations of conjugates against time.

### 3.5. Synthesis of TSPO-Ligand-MTX Conjugates Prodrugs

To a solution of MTX (0.25 g, 0.55 mmol) in anhydrous DMF (2.5 mL) cooled at 0 °C in ice bath was added CDI (0.10 g, 0.68 mmol), and the reaction mixture stirred for 30 min. After this time, a solution of compound **2** (0.306 g, 0.55 mmol) in anhydrous DMF (2.5 mL) was added dropwise, and stirring prolonged for 12 h at room temperature in the dark. Solvent was evaporated under reduced pressure, and the residue dissolved in CH_2_Cl_2_ (20 mL). The resulting solution was washed with 5% NaHCO_3_, dried over Na_2_SO_4_, and rotovapored to dryness. Purification of the residue by silica-gel column chromatography using 15% MeOH/CH_2_Cl_2_ as the eluting solvent, afforded regioisomers **3** (0.34 g, ~70% yield) and **4** (0.1 g, ~25% yield) as orange powders.

5-(2-(2-(4-Chlorophenyl)-3-(2-(dipropylamino)-2-oxoethyl)imidazo[1,2-*a*]pyridin-8-ylamino)-2-oxoethylamino)-4-(4-(((2,4-diaminopteridin-6-yl)methyl)(methyl)amino)benzamido)-5-oxopentanoic acid (TSPO-ligand α-MTX conjugate **3**). m.p. 210 °C (dec.). Anal. calcd. for C_43_H_48_ClN_13_O_6_: C, 58.80; H, 5.51; N, 20.73%. Found: C, 58.94; H, 5.48; N, 20.64%. ^1^H-NMR (DMSO-*d*_6_, 500 MHz): δ 0.80 (t, *J* = 7.5 Hz, 3H, CH_3_), 0.85 (t, *J* = 7.5 Hz, 3H, CH_3_), 1.48 (m, 2H, CH_2_), 1.58 (m, 2H, CH_2_), 1.98 (m, 1H, Glu β-CHH), 2.13 (m, 1H, Glu β-CHH), 2.33 (m, 2H, Glu γ-CH_2_), 3.21 (s, 3H, CH_3_N), 3.23 (m, 2H, CH_2_N), 3.30 (m, 2H, CH_2_N), 4.03 (dd, *J*_1_ = 17.0, *J*_2_ = 6.0 Hz, 1H, Gly α-CHH), 4.10 (dd, *J*_1_ = 17.0, *J*_2_ = 6.0 Hz, 1H, Gly α-CHH), 4.22 (s, 2H, CH_2_CO), 4.50 (m, 1H, Glu α-CH), 4.78 (s, 2H, CH_2_N), 6.62 (br. s, 2H, NH_2_), 6.81 (d, *J* = 9.1 Hz, 2H, Ar CH), 6.90 (m, 1H, Ar CH), 7.04 (br. s, 2H, NH_2_), 7.51 (d, *J* = 8.6 Hz, 2H, Ar CH), 7.70 (d, *J* = 8.6 Hz, 2H, Ar CH), 7.75 (d, *J* = 9.1 Hz, Ar C*H*), 7.96 (m, 2H, Ar CH), 8.16 (d, *J* = 7.5 Hz, Glu NH), 8.38 (t, *J* = 6.0 Hz, Gly NH), 8.56 (s, 1H, Ar CH), 9.82 (s, 1H, NH). HRMS (ESI) *m*/*z* [M − H]^−^: calcd. for C_43_H_47_ClN_13_O_6_^−^ 876.3466. Found 876.3459. IR (KBr): 1698 cm^−1^.

5-(2-(2-(4-Chlorophenyl)-3-(2-(dipropylamino)-2-oxoethyl)imidazo[1,2-*a*]pyridin-8-ylamino)-2-oxoethylamino)-2-(4-(((2,4-diaminopteridin-6-yl)methyl)(methyl)amino)benzamido)-5-oxopentanoic acid. (TSPO-ligand γ-MTX conjugate **4**). m.p. 190 °C (dec.). Anal. calcd. for C_43_H_48_ClN_13_O_6_: C, 58.80; H, 5.51; N, 20.73%. Found: C, 58.94; H, 5.48; N, 20.64%. ^1^H-NMR (DMSO-*d*_6_, 500 MHz): δ 0.80 (t, *J* = 7.5 Hz, 3H, CH_3_), 0.85 (t, *J* = 7.5 Hz, 3H, CH_3_), 1.48 (m, 2H, CH_2_), 1.58 (m, 2H, CH_2_), 2.00 (m, 1H, Glu β-CHH), 2.12 (m, 1H, Glu β-CHH), 2.34 (m, 2H, Glu γ-CH_2_), 3.21 (s, 3H, CH_3_N), 3.23 (m, 2H, CH_2_N), 3.30 (m, 2H, CH_2_N), 4.04 (m, 2H, Gly α-CH_2_), 4.22 (s, 2H, CH_2_CO), 4.31 (m, 1H, Glu α-CH), 4.77 (s, 2H, CH_2_N), 6.60 (br. s, 2H, NH_2_), 6.81 (d, *J* = 9.0 Hz, 2H, Ar CH), 6.90 (m, 1H, Ar CH), 7.42 (br. s, 2H, NH_2_), 7.52 (d, *J* = 8.6 Hz, 2H, Ar CH), 7.68 (d, *J* = 8.6 Hz, 2H, Ar CH), 7.72 (d, *J* = 9.0 Hz, Ar CH), 7.95 (m, 2H, Ar CH), 8.18 (d, *J* = 7.0 Hz, Glu NH), 8.46 (t, *J* = 6.0 Hz, Gly NH), 8.56 (s, 1H, Ar CH), 9.80 (s, 1H, NH). HRMS (ESI) *m*/*z* [M − H]^−^: calcd. for C_43_H_47_ClN_13_O_6_^−^ 876.3466. Found 876.3430. IR (KBr): 1698 cm^−1^.

### 3.6. Biology

#### 3.6.1. Radioligand Binding Assays

Binding assays ([^3^H]Flunitrazepam and [^3^H]PK 11195 bindings) were assessed to membrane preparations from rat cerebral cortex as described previously [15]. The IC_50_ values (GraphPad Prism software, (La Jolla, CA, USA)) were determined from the displacement curves using the reference compound [^3^H]-PK11195, for TSPO or [^3^H]flunitrazepam, for CBR, respectively. The selectivity index (SI) was calculated from: IC_50_(CBR)/IC_50_(TSPO).

#### 3.6.2. Cell Cultures and Cell Viability Analysis by MTT Assay

Rat RG2 and C6 glioma cells were grown in DMEM (Dulbecco’s modified Eagle’s medium, Sigma Aldrich, Milano, Italy) nutrient supplemented with 10% heat inactivated FBS (Fetal Bovine Serum, Sigma Aldrich), 2 mM, l-glutamine, penicillin (100 U/mL), and streptomycin (100 µg/mL) at 37 °C in a humidified 5% CO_2_ atmosphere. Cells were fed every day and seeded in 96-well plates at a density of ~5000 cells/well for assays. MTX and TSPO ligand-MTX conjugates were dissolved in DMSO at initial concentrations of 1000 µg/mL and then diluted with medium. Cells were exposed to different concentrations of compounds (0.1–1000 nM) for 24 h. The percentage of organic solvent (DMSO), used to dissolve the tested compounds d did not exceed 1% (*v*/*v*) in the samples.

Cell viability was evaluated by MTT (3-(4,5-dimethylthiazol-2-yl)-2,5-diphenyltetrazolium bromide) assay as previously described [15]. In brief, 10 µL of MTT (5.0 mg/mL) was added to the cells and cultured at 37 °C, in a humidified 5% CO_2_ atmosphere for 2 h. After this period, medium was removed and the cells were replaced with 150 µL of a DMSO/ethanol (1:1) solution per well. The absorbance of the individual well was measured by microplate reader (Wallac Victor3, 1420 Multilabel Counter, Perkin-Elmer (Waltham, MA, USA). All experiments were repeated three times and each compound’s concentration was tested in triplicate.

#### 3.6.3. Bi-Directional Permeability Study

The bi-directional transport study was conducted as previously reported [15]. A Transwell^®^ 12 plate consisting of differentiated MDCKII-MDR1cells plated on polyester microporous filters (12 mm diameter, 0.4 µm pore size, 0.5 mL apical volume, 1.5 mL basolateral volume) at a density of 100,000 cells/cm^2^, was used for the permeability assay. The integrity of the cell monolayer was assessed by measuring the TEER value in each well, using an epithelial volt-ohm meter (EVOM). Cell monolayers with TEER 120–140 Ohm/cm^2^ were used. In each experiment the cells were equilibrated for 30 min at 37 °C in transport medium which include 0.4 mM K_2_HPO_4_, 25 mM NaHCO_3_, 3 mM KCl, 122 mM NaCl, 10 mM glucose, and the pH was 7.4, with osmolarity of 300 mOsm as determined by a freeze point-based osmometer. For apical-to-basal permeability (AP-BL), test and control compounds solutions were prepared in transport medium at concentration of 75 µM (or 200 µg/mL for FD4), and added to the apical side of the cell monolayer (0.5 mL). Fresh assay medium was placed in the receiver compartment. The transport experiments were carried out in incubator at 37 °C, 5% CO_2_, and 95% humidity. After incubation time of 2 h, samples were removed from the apical side of the monolayer and then analyzed. MTX and TSPO ligand-MTX conjugates **3** and **4** were analyzed by HPLC as described above in the stability studies, while diazepam was analyzed with a PerkinElmer double-beam UV-visible spectromphotometer Lamba Bio 20 (Milan, Italy), equipped with 10 mm path-length-matched quartz cells. The FD4 samples were analyzed with a Victor3 fluorometer (Wallac Victor3, 1420 Multilabel Counter, Perkin-Elmer) at excitation and emission wavelengths of 485 and 535 nm, respectively. All experiments were repeated three times and each compound was tested in triplicate.

### 3.7. Statistical Analysis

The statistical analysis was accomplished using one-way analysis of variance (ANOVA) followed by the Tukey post *hoc* tests (GraphPad Prism version 5.04 for Windows, GraphPad Software, San Diego, CA, USA). Differences were considered statistically significant at *p* < 0.05.

## 4. Conclusions

TSPO ligand-MTX conjugates have shown to possess a high binding affinity and selectivity for TSPO, and exhibited marked toxicity against glioma cells, in comparison with the parent drug MTX. These results also highlight the ability of the TSPO-ligand to transport the hydrophilic drug through biological membranes and determine its accumulation in target cells overexpressing the TSPO. The present work describes the proof-of-concept demonstration of the strength of the bio-conjugate strategy that simultaneously carries inside of cancer cells two agents with distinct modes of action, in the treatment of brain tumors. For this reason, the TSPO ligand-MTX conjugates could be potential tools to increase the effectiveness of the drug in the treatment of brain tumors overexpressing the mitochondrial target TSPO.

## Figures and Tables

**Table 1:** Lipophilicity, permeation of blood-brain barrier (BBB) and stability in phosphate buffer solution and in rat serum solution of TSPO ligand-MTX (methotrexate) conjugates **3** and **4**.

Compound	CLogP ^1^	*t*_1/2_ (h) PBS	*t*_1/2_ (h) Diluted Rat Serum
**2**	+3.25 ± 1.10	-	-
**3**	+5.21 ± 1.37	144 ± 25	4.1 ± 0.9
**4**	+4.40 ± 1.37	74 ± 12	3.3 ± 0.5
MTX	−0.24 ± 0.72	-	-

^1^ Estimated according to ACD/Labs 10 (LogP v.14.04) software (Toronto, ON, Canada); PBS: phosphate buffered saline.

**Table 2:** Affinity for rat cerebrocortical TSPO and central-type benzodiazepine receptors (CBR), cytotoxicity against glioma cells and transport across Madin-Darby canine kidney cells transfected with the human MDR1 gene (MDCKII-MDR1) cells of MTX and TSPO ligand-MTX conjugates **3** and **4**.

Compound	IC_50_ (nM)		SI ^3^	IC_50_ (nM) Rat Glioma Cells		IC_50_ (nM) Human Glioma Cells		Transport across MDCKII-MDR1 ^4^
	TSPO Cortex	CBR Cerebral		C6	RG2	SF126	SF188	*P*_app_ (AP) (cm/s)
**2** ^1^	24.0 ± 4.2	>10^5^	>4.1 × 10^3^	-	-	-	-	-
**3**	7.2 ± 2.1	>10^5^	>1.4 × 10^4^	1.2 ± 0.7 *	4.2 ± 3.2 *	14.2 ± 4.2 *	9.2 ± 3.4 *	3.65 ± 0.14 × 10^−6^
**4**	40.3 ± 3.8	>10^5^	>2.5 × 10^3^	2.2 ± 0.6 *	15.2 ± 2.9 **	25.0 ± 2.6 **	18.2 ± 2.0 **	3.32 ± 0.28 × 10^−6^
MTX	-	-	-	2.6 ± 0.8	3.8 ± 1.5	1.2 ± 0.3	1.2 ± 0.2	9.10 ± 0.44 × 10^−8^
PK11195 ^2^	1.5 ± 1.5	>10^5^	>6.7 × 10^4^	-	-	-	-	-
Flunitrazepam ^2^	-	5.1 ± 0.5	-	-	-	-	-	-

Values are means ± SEM of three experiments performed in duplicate; ^1^ According to [18]; ^2^ According to [5]; ^3^ SI: selectivity index; ^4^ Values are means ± SEM of three experiments performed in triplicate. The statistical differences between TSPO ligand-MTX conjugates **3** and **4**, with respect to MTX was calculated by two-way ANOVA followed by Tukey’s multiple comparison test (* *p* < 0.05, ** *p* < 0.01, *vs.* MTX).

## Abbreviations

BBB: blood-brain barrier
BBTB: blood-brain tumor barrier
CBR: Central-type Benzodiazepine Receptor
CDI: 1,1′-carbonyldiimidazole
CNS: Central Nervous System
COSY: COrrelation SpectroscopY
EDAC: *N*-(3-Dimethylaminopropyl)-*N*′-ethylcarbodiimide hydrochloride
Gly: Glycine
Glu: Glutamic acid
MTX: Methotrexate
NOESY: Nuclear Overhauser Effect SpectroscopY
PBR: Peripheral-type Benzodiazepine Receptor
TEER: Transendothelial electrical resistance
TSPO: Translocator protein (18 kDa)
